# Metabolomics and Fetal-Neonatal Nutrition: Between “Not Enough” and “Too Much”

**DOI:** 10.3390/molecules181011724

**Published:** 2013-09-25

**Authors:** Angelica Dessì, Melania Puddu, Giovanni Ottonello, Vassilios Fanos

**Affiliations:** Neonatal Intensive Care Unit, Puericulture Institute and Neonatal Section, Azienda Ospedaliera Universitaria, Cagliari 09124, Italy

**Keywords:** metabolomics, nutrition, SGA, LGA

## Abstract

Metabolomics is a new analytical technique defined as the study of the complex system of metabolites that is capable of describing the biochemical phenotype of a biological system. In recent years the literature has shown an increasing interest in paediatric obesity and the onset of diabetes and the metabolic syndrome in adulthood. Some studies show that fetal malnutrition, both excessive and insufficient, may permanently alter the metabolic processes of the fetus and increase the risk of future chronic pathologies. At present then, attention is being focused mainly on the formulation of new hypotheses, by means of metabolomics, concerning the biological mechanisms to departure from fetal-neonatal life that may predispose to the development of these diseases.

## 1. Introduction

Metabolomics has been defined as the quantitative measurement over time of the metabolic response of a living system to pathophysiological stimuli and/or genetic modifications [1]. This discipline, based on the use of mathematical and statistical methods to solve multivariate problems, is capable of describing the chemical profile of a biological system in terms of low-molecular-weight metabolites present in cells, tissues, organs and biological fluids. By means of a metabolic approach it is thus possible “in real time” to photograph influences on the organism such as biochemical perturbations caused by diseases, drugs and toxins, thus delineating the biochemical phenotype of a biological system [2]. In the medical field, attention is focusing on the definition at the biochemical and molecular levels of the different pathophysiological states that characterize human beings in the period that goes from embryonic development to ageing and death. The goal of this characterization is to develop “customized” therapeutic approaches for each individual. At present, the literature is focusing on new hypotheses concerning the biological mechanisms that lead to infantile obesity and the onset of diabetes and the metabolic syndrome in adulthood [3]. Experimental studies show that fetal malnutrition, both excessive and insufficient, may permanently alter the metabolic processes of the fetus and increase the risk of future chronic diseases. In this field the metabolomic studies in the literature appear promising in that they provide a sharper and more complete picture of the biochemical and molecular state in the pre- and post-natal period. They may be capable of revealing which metabolic alterations induced in the fetal period can be addressed to avoid the onset of diseases in adulthood.

## 2. How Big Is the Problem?

Cardiovascular diseases are the most important causes of death in all the World; every year 4.3 million people die of them in Europe and they account for 48% of all deaths (54% women and 43% men). Obesity and diabetes are also among the most important risk factors for ischemic cardiopathy and it is estimated that approximately 48 million adults in the European Union suffer from diabetes. Also to be considered is the fact that both the pathologies are on the increase [4]. Infantile obesity is also on the rise constantly and in many European countries one child out of five suffers from obesity or is overweight. A cause for concern is represented by the persistence of infantile obesity into adulthood, with the consequent increase in health risks [5]. In recent years, the coming to the fore of genomics and proteomics in the field of metabolic diseases has shown that infantile obesity and adult metabolic syndromes are closely connected by both genetic and environmental factors. In the literature we find many works that associate genetic polymorphisms with metabolic diseases such as diabetes and obesity both in adults and children [6,7,8]. However, relatively little is known about the mechanisms by means of which most gene loci act. Also poorly understood are the mechanisms by means of which the environment and a diet rich in fats, together with a sedentary lifestyle, may increase the risk of developing metabolic diseases [9].

## 3. The Foetal Hypothesis: Everything Begins at the Beginning

The first scholar who hypothesized the foetal origin of certain chronic diseases of the adult was Barker [10,11]. This author suggested that changes in the intrauterine and early postnatal metabolic-nutritional environment impact on the basic vital processes and increase the risk of developing long-term diseases. This phenomenon, known as “perinatal programming” refers to intrauterine or perinatal events in a critical period of development for different organs and tissues that may negatively influence their proper functioning, with the possibility of making an individual more susceptible to contracting certain diseases than other. Then, the intrauterine environment defines the epigenetic profile of newborns, with implications for the risk of developing diseases later in adult life. This means that the programming of cardiovascular risk and obesity in adulthood takes place starting from intrauterine life. Barker suggested that there was a relationship between low birth weight and the increased risk of contracting cardiovascular diseases. This theory is now accepted by most members of the academic community, not lastly because after Barker several other studies have found an association between low birth weight and the risk of presenting a metabolic syndrome and type 2 diabetes in adulthood. This theory postulates that the underfed foetus activates a series of adaptive mechanisms to increase the immediate possibility of survival such as the storing of glucose to ensure nutrients to the most vital organs, with a consequent reduction in insulin secretion that continues in postnatal life, thus increasing the risk of obesity in later life [12]. However, some researchers have found that not only low birth weight, but also high birth weight, as in the case of diabetic mothers, is followed by an increased risk of developing type 2 diabetes in adulthood [13]. Harder *et al*. performed a meta-analysis [14] on the relationship between low/high birth weight and the onset of type 2 diabetes. Some of these studies provided estimates which were adjusted for gestational age. The authors found a nonlinear, U-shaped association between birth weight and type 2 diabetes which led to an augmented risk both for high birth weight (LGA)(OR = 1.27) and low birth weight (SGA) neonates with respect to their gestational ages. This emphasizes that high birth weight was found to be associated with increased risk of type 2 diabetes in later life to the same extent as low birth weight.

## 4. SGA & LGA: Are They Different?

**Figure 1:**
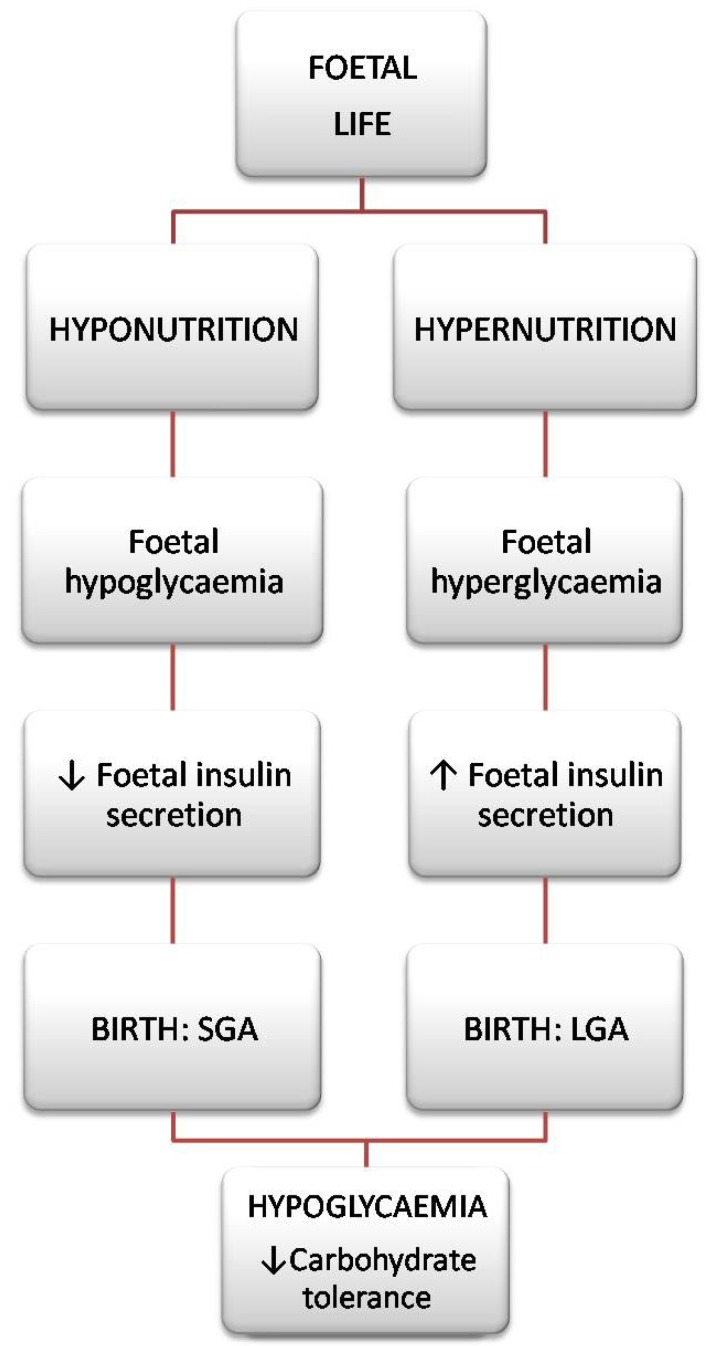
Metabolic state characterizing SGAs and LGAs from foetal life to birth.

Traditionally the definition of SGA is an infant that has a birth weight below the 10th centile for gestational age. It is known as the low birth weight is in itself closely related to infant morbidity and mortality. In addition to prematurity, intrauterine growth retardation (IUGR), is considered one of the most important causes of low birth weight [15]. According to the classification of the American College of Obstetricians and Gynaecologists, IUGR is defined as a fetus that does not reach its growth potential and is characterized at birth by a weight or a body mass index below normal for the number of gestational weeks [16]. It should be emphasized that not all SGA infants identified at birth are patients with IUGR, as well as may be infants with IUGR are not classified as SGA. Among the many causes of IUGR, there are universally recognized risk factors such as cigarette smoking, maternal malnutrition and vascular/placenta disease [17]. It’s instead defined LGA or macrosoma as a neonate with a birth weight above the 90th centile for gestational age. Among the major risk factors for macrosomia include: maternal diabetes, obesity and excessive nutrition/weight gain during pregnancy [18]. Therefore it can be affirm that the intrauterine environment is the main factor that affects the growth and fetal development [19]. In fact, fetal life is a period characterized by phases of rapid cell proliferation and differentiation during which a factor damaging to the environmental (epigenetic) may cause permanent effects such as structural and functional alterations of organs, a reduction in the number of cells and a different adjustment of the hormonal axes. However, it is interesting to note that there are significant analogies between neonates with growth retardation and those of high birth weight. Firstly, macrosomia in LGA is selective as is microsomia in SGA: what is striking is the subcutaneous fat, the liver and the spleen, while the brain is safeguarded. The cranial circumference is normal, but there is a clear disproportion between this and the abdominal circumference (inversely proportional depending on whether it is a case of SGA or LGA). Moreover, despite the different foetal nutrition that accompanies the two pathologies, in both cases we can find quite similar complications at birth. In the case of a foetus with intrauterine growth retardation we can hypothesize that a reduced carbohydrate amount may cause a reduction in insulin secretion, while in the LGA foetus there will be too much insulin caused by the hyperglycaemic condition it undergoes [20]. In any case, at birth the LGA baby often undergoes hypoglycaemia caused by chronic foetal hyperglycaemia which continues even after birth. The same complication frequently affects SGA neonates who maintain their hypoglycaemic condition after birth. Several studies on animal models have confirmed that exposure to a hyper- and hypoglycaemic environment in the uterus leads to reduced carbohydrate tolerance at birth which continues into adulthood, independently of subjects’ genetic predisposition [21,22]. This underscores the importance of epigenetics over genetics in the role of maternal-placental-foetal transmission. It would thus appear that SGA and LGA neonates, despite the opposing metabolism that characterizes them during their foetal life, find at birth a common condition of reduced carbohydrate tolerance that tends to persist during growth and into adulthood, consequently exposing them to a higher risk of developing pathologies such as obesity and type 2 diabetes (Figure 1). 

Another important element in the pathogenesis of a metabolic syndrome appears also to be neonatal overfeeding: the results of some authors [23] indicate that overfeeding in the neonatal period may lead to a “*malprogramming*” of the neuroendocrine circuits of the mediobasal hypothalamus that control appetite, body weight and the metabolism. It is also true that at present the nutritional practice in nurseries and neonatal intensive care units is to quickly recover the birth weight of infants, with the result that both SGAs and LGAs are fed to this end. The same can be said in the case of those with hypoglycaemia at birth: they receive the same therapy based on glycaemia values.

## 5. Metabolites and Metabolomics in SGA and LGA

Also as concerns the single metabolites, there appear to be important analogies between SGAs and LGAs, first and foremost those of leptin. Leptin, a hormone produced by adipose tissue, acts at the level of the hypothalamus to limit appetite and lipogenesis. It is capable of activating the mammalian target of rapamycin (mTOR) kinase which by regulating cell growth is essential in mediating leptin action in the hypothalamic circuits involved in regulating the body energy balance. In the blood of obese individuals we find high levels of leptin not associated with a reduced food intake: in such cases we speak of “leptin resistance” [24]. This can be attributed to genetic causes (lack of the receptor, lack of transducers of the leptin signal), but in most cases the cause cannot be identified exactly. The same resistance phenomenon takes place in type-2 diabetics who, although they produce large amounts of insulin, are still hyperglycemic and thus insulin-resistant. Insulin and leptin resistance often go hand in hand and may coexist in the same patient. It must also be kept in mind that the placenta is capable of producing a significant leptin amount and also insulin treatment increase the production of leptin by the placenta and as a result fetal circulating leptin. Such phenomena may be important owing to their long-term consequences for intrauterine growth and future energy homeostasis. Chronic fetal hyperleptinemia and accelerated fetal growth accompanied by increased amounts of body fat are frequent findings in the offspring of diabetic or obese mothers [25]. It is interesting to note that even low-birth-weight neonates have been associated with increased leptin values in the first months of life [26]. A study conducted on IUGR rats has demonstrated that chronic placental ischemia results in numerous alterations of the fetal environment that contribute to the development of impaired glucose metabolism, insulin resistance and hyperleptinemia in young offspring [27]. Recently, thanks to new metabolomic analysis techniques, studies have been performed to provide a more global vision of the metabolic profiles that characterize SGAs (Table 1). Only preliminary NMR spectroscopy data from the study by Logan *et al*. [28] have shown promising results in neonates of diabetic mothers. In comparison to a small number of newborn healthy term control infants, the urinary metabolic profiles of infants of diabetic mothers appeared to differ (Table 1). However, another work has been published in which changes in the serum metabolome were assessed prospectively in children who later progressed to type 1 diabetes [29] were assessed. The authors demonstrate that the appearance of insulin and glutamic acid decarboxylase autoantibodies was preceded by diminished ketoleucine and elevated glutamic acid. Recently, the same authors performed a study on non-obese pre-diabetic mice which recapitulated the design of the human study and derived the metabolic states from longitudinal lipidomics data. They showed that metabolomics confirmed dysregulation of energy and the amino acid metabolism in the islets of high-risk mice: in particular several key metabolites of these pathways were found to be upregulated, including glutamic and aspartic acids [30]. It is interesting to note that the metabolites in SGAs and in those of the children of diabetic mothers as well as in children who later developed diabetes are closely correlated and all belong to the same metabolic cycle involved in the tricarboxylic acid (TCA) cycle. It is known that insulin plays an important role in energy generation through oxidation of acetyl-CoA and by favoring the conversion of glucose into piruvate in the TCA cycle. Insulin resistance or glucose intolerances may therefore lead to altered TCA cycle intermediates [31]. Thus it is probable that both the SGAs and the children of diabetic mothers showed a similar metabolic pattern for the very reason that they were both in a condition of reduced sensitivity to insulin at birth. In any case, it will be necessary in the future to uphold these results with further studies so as to arrive at a broader picture from the metabolic viewpoint of the two pathologies and, with the help of metabolomics, to compare the metabolic phenotype of SGAs and LGAs.

**Table 1:** Metabolomic studies that analyze the metabolic profiles of SGAs and LGAs.

Author	Population study	Sample	Metabolomic analysis	Metabolites results
**van Vliet *et al*. 2013 [32]**	10 IUGR *vs*. 6 control rabbit fetuses	Brains	LC-QTOF-MS	Aspargine, ornithine, N-acetylaspartylglutamic acid, N-acetylaspartate and palmitoleic acid
**Lin *et al*. 2012 [33]**	18 IUGR *vs*. 18 control pig fetuses	Umbilical vein plasma	Q-TOF MS	Pyroglutamic acid, carnitine and creatinine
**Favretto *et al*. 2012 [34]**	22 IUGR *vs*. 21 control human neonates	Cord blood	LC-HRMS	Phenilalanine, tryptophan acid and glutamate
**Logan *et al*. 2012 [28]**	18 IDM *vs*. 12 healthy term control infants	Urine	NMR	Glucose, formate, fumarate, succinate and citrate
**Dessì *et al*. 2011 [35]**	26 IUGR *vs*. 30 control human neonates	Urine	NMR	Myo-inositol, sarcosine, carnitine and creatinine
**Horgan *et al*. 2011 [36]**	8 IUGR *vs*. 6 control human neonates	Venous cord plasma	UPLC-MS	Phenylacetylglutamine, carnitine, hydroxybutyrate
**Nissen *et al*. 2011 [37]**	12 IUGR *vs*. 12 control newborn piglets	Plasma	NMR	Myo-inositol and D-chiro-inositol

## 6. Conclusions

Metabolomics can be considered a valid, noninvasive instrument in the study of the components produced by the metabolism even in fetal and neonatal life. The studies published to date represent a starting point of fundamental importance in assessing the metabolic correlations between SGAs and LGAs for the purpose of identifying the single metabolites that characterize them. Feeding, as well as therapy in cases of hypoglycemia, should be customized and adapted to individual needs for both SGAs and LGAs, keeping in mind their long-term consequences. Data in the most recent publications suggest that by means of metabolomics it will be possible in the immediate future to monitor such patients over time to prevent the onset of chronic diseases in adulthood.
